# The Conformational Change of the L3 Loop Affects the Structural Changes in the Substrate Binding Pocket Entrance of β-Glucosidase

**DOI:** 10.3390/molecules28237807

**Published:** 2023-11-27

**Authors:** Ki Hyun Nam

**Affiliations:** College of General Education, Kookmin University, Seoul 02707, Republic of Korea; structure@kookmin.ac.kr

**Keywords:** β-glucosidase, loops, conformational change, substrate-binding pocket entrance, biomass-degrading

## Abstract

β-glucosidase (Bgl) hydrolyzes cellobiose to glucose, thereby releasing non-reducing terminal glucosyl residues. Bgl is an essential enzyme belonging to the biomass-degrading enzyme family, which plays a vital role in enzymatic saccharification during biofuel production. The four loops above the Bgl substrate-binding pocket undergo a conformational change upon substrate recognition. However, the structural dynamism of this loop and how it is conserved among Bgl family members remain unknown. Herein, to better understand the four loops above the substrate-binding pocket of Bgl, four Bgl crystal structures in *Thermoanaerobacterium saccharolyticum* (TsaBgl) were determined at 1.5–2.1 Å. The L1, L2, and L4 loops of TsaBgl showed a rigid conformation stabilized by their neighboring residues via hydrogen bonds and hydrophobic interactions. The TsaBgl L3 loop showed relatively high flexibility and two different N-terminal region conformations. The conformational change in the TsaBgl L3 loop induced a change in charge and shaped at the substrate-binding pocket entrance. The amino acid sequences and structures of the TsaBgl L1–4 loops were compared with other 45 Bgl proteins, and a diversity of the L2 and L3 loops was observed. Differences in amino acids and lengths of Bgls L2–L3 loop induced differences in the conformation and structure of the Bgls substrate-binding pocket entrance. These findings expand our knowledge on the molecular function of the loops in the Bgl enzyme family.

## 1. Introduction

β-glucosidases (Bgls, β-d-glucopyranoside glucohydrolases, E.C. 3.2.1.21) cleave β-1,4-glycosidic bonds in disaccharides or glucose-substituted polysaccharides, thereby releasing non-reducing terminal glucosyl residues [1,2,3]. This enzyme family is ubiquitous and is found in all living organisms, including archaea, eubacteria, and eukaryotes [4]. It is involved in diverse cellular functions, including biomass conversion in bacteria and fungi, breakdown of glycolipids and exogenous glucosides in animals, lignification as a defense mechanism against cell wall oligosaccharide catabolism by phytopathogens, other defense mechanisms, activation of phytohormone conjugates, and scent release in plants [5,6]. Furthermore, Bgl is involved in both plant–microorganism and plant–insect interactions [6]. Bgl is also used in several biotechnological processes, including the development of new carbohydrate foods and other commercial products from cellulose [7,8,9]. Moreover, this enzyme is essential for producing glucose molecules during ethanol and biofuel production using biomass [10,11].

Bgl proteins have a TIM-barrel fold [12,13,14]. At the center of this TIM-barrel fold, there is a substrate-binding pocket that is accessible to substrate-linear sugar molecules. The substrate-binding pocket can be divided into the glycone, aglycone, and gatekeeper regions; in addition, the bound sugar substrate is cleaved via a retention mechanism using two glutamate residues [6,15]. The gating region is known to be involved in substrate specificity and glucose tolerance. Four loops are present above the Bgl substrate-binding pocket, forming a large cavity for the nascent substrate to access [16]. It has been proposed that these loops at the Bgl entrance change conformation when a substrate approaches them [16,17]. However, the structural and conformational characteristics of this change, as well as the detailed structural analysis of the degree of flexibility exhibited by the Bgl loops, remain unknown.

We previously characterized Bgl from *Thermoanaerobacterium saccharolyticum* (TsaBgl) and determined its crystal structure [18]. TsaBgl showed maximal hydrolase activity at pH 6.0 and a temperature of 55 °C on *p*-nitrophenyl-β-d-glucopyranoside. This enzyme exhibited tolerance to glucose, a known product inhibitor of Bgl. The glucose tolerance-related gatekeeper region in the substrate-binding site contained the unique amino acid residues, Leu170 and Trp323. The β8-α14 loop at the entrance of the TsaBgl substrate-binding site showed a relatively higher B-factor value than other amino acid sites and exhibited a unique conformation as compared to that of other Bgls. Similar structural analyses have been carried out for the loop conformation in other Bgls [9,16]. Therefore, we hypothesized that the conformation of the β8-α14 loop in TsaBgl may vary despite the absence of structural evidence to support this. Particularly, Bgl loops are closely related to enzyme activity in terms of substrate access and recognition. Therefore, understanding the structural characteristics and flexibility of Bgl loops and comprehensively analyzing the structure activity characteristics of the Bgl family may provide important insights for improving the efficiency of the industrially applied Bgl enzyme. However, the involvement of structural changes in these loops in Bgl substrate recognition has not yet been determined.

In this study, to better understand the four loops above the Bgl substrate-binding pocket, we performed an expanded crystallography study on TsaBgl as a model protein and determined the crystal structures of four TsaBgls from two different crystal forms. The effects of the structural properties of the four loops on the TsaBgl substrate-binding pocket were analyzed. Subsequently, structural changes in the substrate-binding pocket induced by conformational changes in the L3 loop were analyzed. The amino acid sequences and structures of the four loops on the substrate-binding pockets of 46 Bgls were comprehensively compared. Overall, this study aimed to broaden knowledge on the four loops on the Bgl substrate-binding pocket.

## 2. Results

### 2.1. Structure Determination

To investigate whether the loops on the TsaBgl substrate-binding pocket had different conformations, as well as the structural effects caused by this change, an expanded crystallization experiment was performed. Two different space groups of TsaBgl crystals containing triclinic P1 and orthorhombic P2_1_2_1_2_1_ were obtained at pH 7.5 and 8.0, respectively. P1 is a new TsaBgl crystal form containing the four molecules in an asymmetric unit, whereas P2_1_2_1_2_1_ has similar unit cell parameters as previously reported TsaBgl crystal structures (PDB code: 7E5J) [18]. Two diffraction datasets, each for P1 and P2_1_2_1_2_1_, were collected to verify the flexibility of the TsaBgl loops (Table 1). Two TsaBgl-P1 structures were processed up to 1.9 and 2.1 Å, with R_work_/R_free_ of 0.1694/0.1952 and 0.1599/0.2019, respectively. Two TsaBgl-P2_1_2_1_2_1_ structures were processed up to 1.5 and 1.6 Å, with R_work_/R_free_ of 0.1546/0.1712 and 0.1506/0.1762, respectively. The electron density maps of TsaBgl-P1 and TsaBgl-P2_1_2_1_2_1_ structures were clearly observed (Supplementary Figure S1), except for the partially disordered electron density maps between the Gly302 and Gly304 residues in TsaBgl-P1 (chain C and D for Data I and II, respectively).

**Figure 1:**
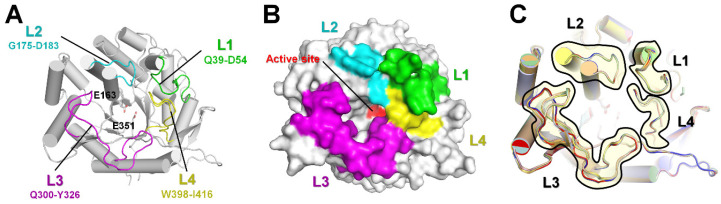
Crystal structure of TsaBgl. (**A**) Cartoon and (**B**) surface structure representations of TsaBgl. The L1, L2, L3, and L4 loops are indicated by green, cyan, pink, and yellow, respectively. Catalytic residues are indicated by sticks or red surface. (**C**) Superimposition of TsaBgl molecules obtained from P1 and P2_1_2_1_2_1_ crystals.

All TsaBgl structures in the P1 and P2_1_2_1_2_1_ crystal forms exhibited the typical (β/α)_8_ TIM-barrel fold (Figure 1A). There were four loops, L1 (Qln39-Asp54), L2 (Gly175-Asp183), L3 (Gln300-Tyr326), and L4 (Trp398-Ile416), above the TsaBgl substrate-binding site. These loops formed a pocket on the substrate-binding site. The L2 and L4 loops were directly above the substrate-binding pocket, and the L1 loop was located above the L2 and L3 loops (Figure 1A). The L3 loop had the longest amino acid chain; its N-terminus (Qln300-Val310) was farthest from the active substrate-binding pocket, and its C-terminus (Glu320-Ile325) was close to the substrate-binding site. The TsaBgl substrate-binding pocket was surrounded by the L1–L4 loops without the space between the L2 and L3 loops (Figure 1B). Superimposition of the TsaBgl structures showed a significant conformational change in the L3 loop region, whereas the L1, L2, and L4 loops showed almost similar conformations (Figure 1C). Particularly, the L3 loop exhibited two structurally distinct conformations, one where it was oriented towards the substrate-binding pocket (folded L3 loop) and the other where it was oriented towards the solvent direction, i.e., away from the substrate-binding site (straight L3 loop) (Figure 1C). These differences in the conformation of the L3 loop induced differences in the structure of the TsaBgl substrate-binding pocket (see below). The TsaBgl substrate-binding site can be divided into the glycone, aglycone, and gatekeeper regions. A Tris molecule was shown to act as an inhibitor of TsaBgl β-glucosidase activity [18]. This molecule was found to bind to all the active sites in the TsaBgl glycone region. All Tris molecules on TsaBgl were coordinated by Gln18, Glu163, Glu351, Glu405, and Trp406, and the orientations of these Tris molecules in the TsaBgl active site pocket were identical to those previously reported [18]. Superimposition of TsaBgl TIM-barrel folds, excluding the loop regions, showed r.m.s. deviations ranging from 0.037 to 0.184 Å. These results indicated that the conformation of the L3 loop had no effect on the structure of the TsaBgl substrate-binding pocket.

### 2.2. Structural Dissection of Loops above the TsaBgl Substrate-Binding Pocket

**Figure 2:**
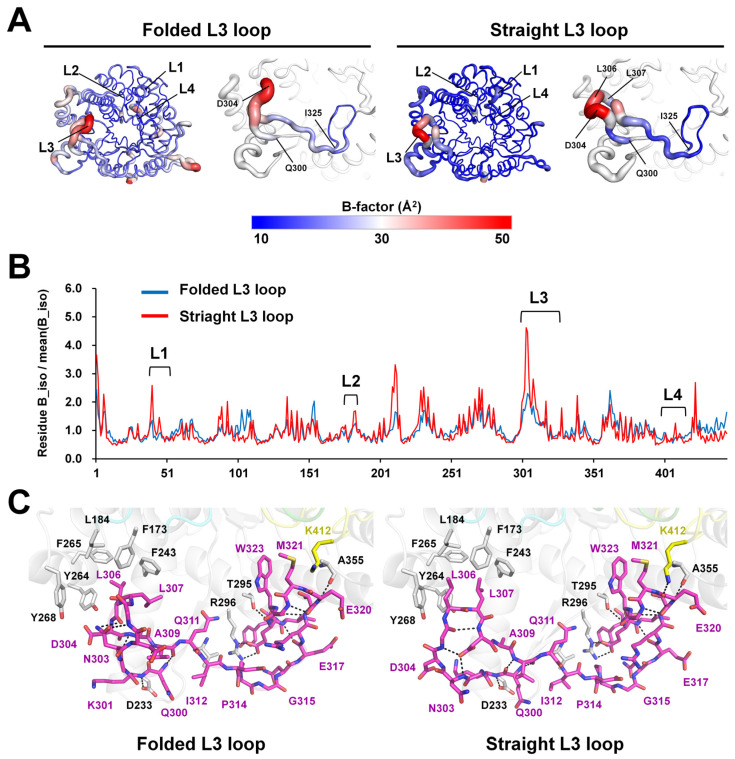
Structural analysis of the loops above the TsaBgl substrate-binding pocket. (**A**) B-factor putty representation of TsaBgl molecules for Data I (chain A: folded L3 loop) and Data III (Chain A, straight L3 loop). (**B**) Plot showing normalized B-factor values of Cα atoms in the folded (Data I, chain A) and straight (Data III, chain A) L3 loop conformations. (**C**) Interaction of the folded (Data I, chain A) and straight (Data III, chain A) L3 loops with neighboring residues. Hydrogen bonds are indicated using black dotted lines.

The B-factor putty representation showed that the L1, L2, and L4 loops had rigid conformations, while the long L3 loop was found to exhibit flexibility with two main conformations (Figure 2A and Figure S2). Superimposition of the TsaBgl L1, L2, L3, and L4 loops showed r.m.s. deviations ranging from 0.030 to 0.253, from 0.038 to 0.126, from 0.049 to 0.825, and from 0.031 to 0.132 Å, respectively. In addition, the B-factor analysis showed that the B-factor values of the L1 and L2 loops were similar to those of the whole protein (Figure 2B, Supplementary Figure S2 and Table S1). Conversely, the B-factor value of the L3 loop was higher than that of the whole protein, indicating that this region had higher flexibility than the other TsaBgl regions (Figure 2A, Supplementary Figure S2, and Table S1). Particularly, the L3 loop N-terminal (Qln300-Val310) showed high flexibility, while its C-terminal (Glu320-Ile325) exhibited a rigid conformation (Figure 2A). Superimposition of the L3 loop N- and C-terminals in all TsaBgls showed r.m.s. deviations ranging from 0.047 to 2.825 and from 0.018 to 0.169 Å, respectively. In contrast, the L4 loop showed a lower B-factor value than the whole protein, indicating that it was rigid (Figure 2A, Supplementary Figure S2 and Table S1).

**Figure 3:**
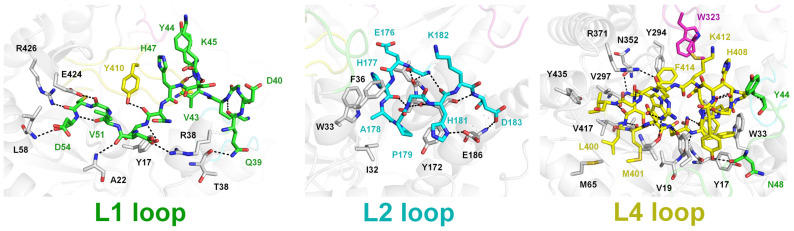
Interactions of the L1, L2, and L4 loops with neighboring residues in the TsaBgl molecule. Amino acids in the L1, L2, L3, and L4 loops are indicated using green-, cyan-, pink-, and yellow-colored sticks, respectively. Hydrogen bonds are indicated using black dotted lines.

To understand the structural rigidity and flexibility of loops on the TsaBgl substrate-binding pocket, intra-molecular interactions of the L1, L2, L3, and L4 loops were analyzed using the chain A molecule from Data I and Data III. The L1 loop exhibited a rigid conformation stabilized by hydrogen bonds (Gln39-Lys42, Val43-Ser46, Gly49-Tyr17, Asp50-Arg38, Asp50-Ala22, Val151-Asp54, Cys53-Arg426, and Asp54-Leu58) (Figure 3). Moreover, the Tyr44 and Asn48 residues of the L1 loop interacted with the Ala407 and Tyr410 residues of the L4 loop, respectively (Figure 3). The rigid conformation of the L2 loop was also stabilized by hydrogen bonds (Gly175-Leu170, Glu176-Leu170, Gly180-Ser171, His181-Ser171, His181-G175, and Asp193-Tyr172) (Figure 2). Furthermore, the side chain of the Ala178 residue was stabilized by hydrophobic interaction with Ile32, Trp33, and Phe36 (Figure 3). The two different conformations of the straight and folded L3 loops showed distinct interactions with neighboring amino acids in the flexible N-terminal region, whereas the rigid C-terminal region of the L3 loop showed almost identical interactions with neighboring residues (Figure 2C). In the folded L3 loop, the Leu307 residue was stabilized by hydrophobic interactions with Ile239, Phe243, Tyr264, and Phe265; whereas in a straight L3 loop (Data III, chain A), the Leu306 and Leu307 residues were stabilized by hydrophobic interactions with Phe173, Leu184, Ile239, Phe243, Tyr264, and Phe265 (Figure 2C). These findings indicated that the conformation of the folded L3 loop in TsaBgl was stabilized by extended hydrophobic interactions. The straight and folded TsaBgl L3 loop conformations exhibited common hydrogen bonds (Glu317-Lys370 and Glu320-Ala355) and hydrophobic interactions (Ile312-Ala227, Ile312-Val298, Met321-Phe414, Trp323-Tyr294, Ile325-Phe256, and Ile325-Phe374). The L4 loop exhibited compact folding and was buried with neighboring residues. It was stabilized by hydrogen bonds (Leu400-Ala13, Asn403-Tyr421, Ala407-Tyr44, and Lys412-Glu320) and hydrophobic interactions (Leu400-Met66, Met401-Met65, Phe404-Tyr17, Phe404-Trp33, Trp406-Tyr17, Trp406-Trp33, Trp406-Trp119, Ile416-Val12, Ile416-Leu375, Ile416-Val397, and Ile416-Tyr435) (Figure 3).

The L1, L2, and L4 loops exhibited a rigid conformation due to their multiple hydrogen bonds and hydrophobic interactions with neighboring amino acids. In contrast, the L3 loop interacted less with neighboring amino acids than the other loops, resulting in a higher overall relative B-factor value. Particularly, in the L3 loop, mobility was observed at the N-terminus. In the folded loop, the Leu306 residue maintained hydrophobic interactions with the surrounding hydrophobic residues, but the Leu307 residue did not interact with surrounding amino acids. In contrast, in the straight L3 loop, both the Leu306 and Leu307 residues maintained hydrophobic interactions with surrounding hydrophobic residues. This finding indicated that the N-terminal Leu306 and Leu307 residues of the L3 loop did not maintain very stable hydrophobic interactions. Particularly, as both the folded and straight L3 loop conformations were observed within a single crystal in Data I and II, this indicated that the two conformations can appear in the same environment.

### 2.3. L3 Loop Conformation-Induced Structural Differences

**Figure 4:**
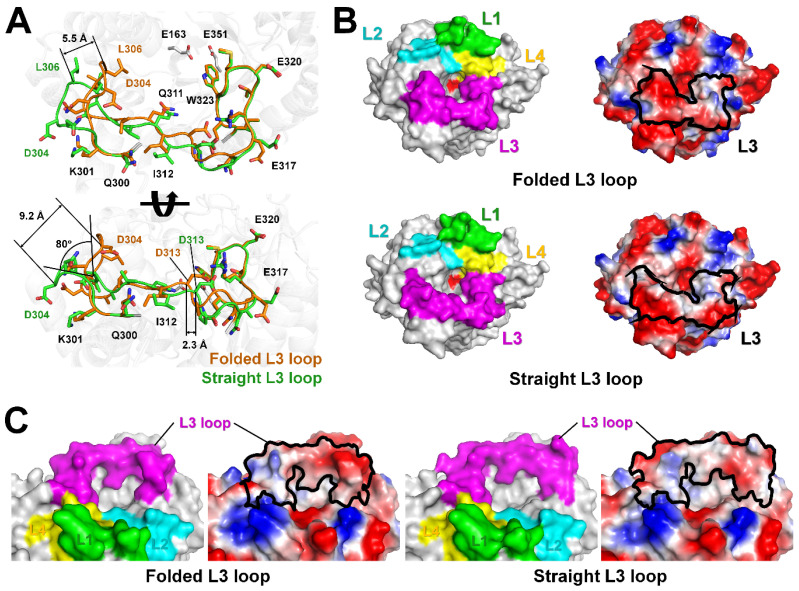
Analysis of the conformation of the L3 loop in TsaBgls. (**A**) Superimposition of the folded (Data I, chain A) and straight (Data III, chain A) TsaBgl L3 loops. (**B**) Top-view and (**C**) side-view of the surface and electrostatic surface structures of the folded (Data I, chain A) and straight (Data III, chain A) TsaBgl L3 loop conformations.

The L3 loop region, which interacted less with neighboring residues, exhibited structurally distinct conformational differences. We analyzed the effects of these structural changes in the L3 loop on the structural integrity of TsaBgl. In the P1 and P2_1_2_1_2_1_ crystals, the L3 loop regions of the TsaBgl-Data I (folded L3 loop conformation) and TsaBgl-Data III (straight L3 loop conformation) chain A molecules, which showed the most significant differences in L3 loop conformation, were carefully analyzed. Superimposition of these TsaBgl molecules showed an r.m.s. deviation of 0.134 Å. Among the 26 amino acids (Gln300-Ile325) comprising the L3 loop, a different main-chain position was observed between the Lys301 and Arg317 residues. Particularly, the L3 loop in the structure between the K301 and Q311 residues showed two different straight or folded conformations (Figure 4A). While the straight L3 loop had a relatively straight loop structure in the direction pointing away from the substrate-binding pocket, the folded L3 loop was oriented toward the substrate-binding pocket. Between the straight and folded L3 loops, the Gly303 residue was rotated by approximately 80°. The distances between Cα atoms of the Asp304 and Leu306 residues of the straight and folded L3 loops were approximately 9.2 Å and 5.5 Å, respectively. In addition, a positional shift of approximately 2.3 Å in the distance of the Cα atom of the Asp313 residue was observed between the straight and folded L3 loops around the substrate-binding pocket. In the top view of the TsaBgl substrate-binding site, the L3 loop in the folded conformation induced a greater narrowing of the substrate-binding pocket than the straight L3 loop and exhibited a relatively negatively charged surface (Figure 4B). In the side view of the TsaBgl substrate-binding pocket entrance, the folded L3 loop displayed a relatively negatively charged surface but exhibited a similar non-polar surface near the entrance (Figure 4C). The substrate accessible width of the substrate-binding pocket entrance with the folded L3 loop conformation was approximately 30 Å, and the proximate distance between the L2 and L3 loops was approximately 5 Å. Conversely, the substrate accessible width of the substrate-binding pocket entrance with the straight L3 loop conformation was approximately 35 Å, and the proximate distance between the L2 and L3 loops was approximately 7 Å. We speculated that the straight L3 loop conformation made the substrate entrance wider, thereby providing easier access to the substrate than the folded conformation.

### 2.4. Analysis of the Loop Region in Bgl Family Enzymes

Previous structural analyses have shown diverse loop conformations at the active site pocket of Bgls [9]. However, to the best of our knowledge, a detailed analysis of the characteristics of Bgl amino acid sequences and structures has not been reported. To better understand the loop conformations above the Bgl substrate-binding pocket, we analyzed the amino acid sequences and structures of the L1–L4 loops of 46 Bgl species using data derived from bacteria, archaea, eukaryote, and unclassified organisms deposited in the Protein Data Bank (Table S1). Phylogenic tree data showed that the Bgls three-dimensional structure determined for bacteria, archaea, and eukaryotes showed closeness between each class (Supplementary Figure S3). Data of unclassified Bgl from uncultured microorganism (unmicroBgl, A0A1E1FFN6) and unidentified (unidBgl, A0A2I2LGB3) on CAZY were evolutionally close to SspBgl and ManBgl, respectively, which belong to the bacterial Bgl class (Supplementary Figure S3).

Amino acid similarities between the four loops on the Bgl substrate-binding site were analyzed (Figure 4). The L1 loop of Bgls comprised 11–18 amino acids. When compared to the TsaBgl amino acid sequence, which comprises 14 amino acids, archaea PfuBgl and TasBgl were found to have four amino acid insertions, while PhoBgl and MspBgl were found to have two and one less amino acid, respectively. Absolute conserved Bgl L1 loop amino acids were not observed. The L2 loop of Bgls comprised 8–26 amino acids, and MspBgl and unbacBgl3 had 26 and 24 amino acids more than TsaBgl, which had nine amino acids, respectively. Although some Ala-Pro-Gly amino acid residues were mostly conserved in the L2 loop of Bgls, there was no overall amino acid similarity. The L3 loop of Bgls was composed of 20–48 amino acids (Supplementary Figure S4). The number of amino acids in the L3 loops of Bgls from the archaea and eukaryote classes was higher than that of the L3 loops of Bgls from the bacterial class, excluding ManBgl, SspBgl, and unbacBgl2. The L4 loop of Bgls comprised 19 amino acids, with no differences in the number of amino acids. In addition, eight amino acids (WxxxDNxEWxxGxxxxFG) were conserved in the L4 loop. In summary, the Bgl L4 loop of different species showed high similarity in terms of amino acid length and sequence conservation, but the L1, L2, and L3 loops did not show this similarity.

**Figure 5:**
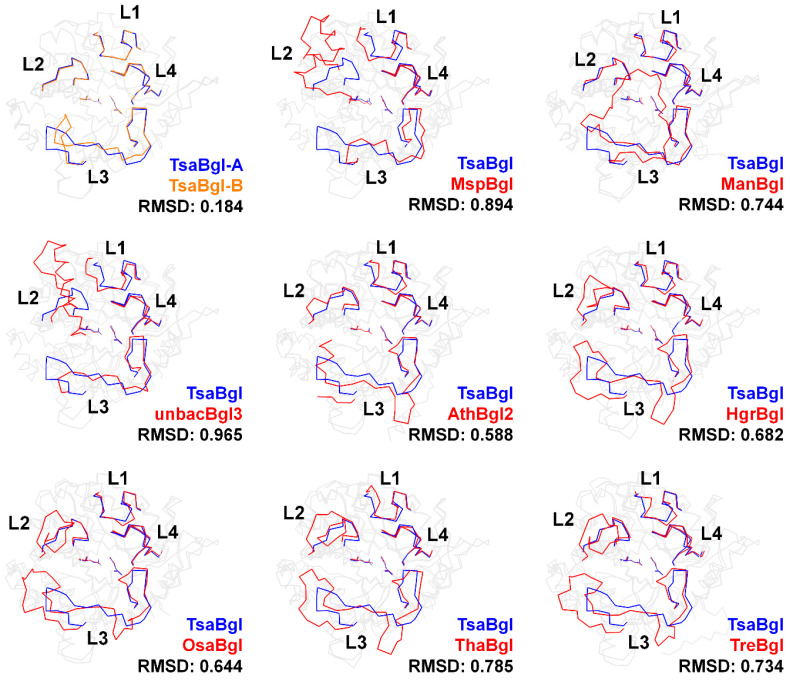
Structural comparison of the loop structures on the substrate-binding pocket of TsaBgl with that of MspBgl (PDB code: 4R27), ManBgl (3W53), unbacBgl3 (6IER), AthBgl2 (5OGZ), HgrBgl (4MDO), OsaBgl (3GNO), ThaBgl (5JBO), and TreBgl (1CBG).

To better understand the loop structure, the crystal structure of the four loops in TsaBgl was compared with the crystal structures of 48 other Bgls (Figure 5 and Supplementary Figure S5). The conformation of the TsaBgl L1 loop was similar to that of the L1 loops of most Bgls, excluding that of PfuBgl, PhoBgl, TagBgl, NkoBgl, PsaBgl2, and SfuBgl. The slightly different conformation of the TsaBgl L1 loop with respect to that of other Bgls was due to the influence of the different number of amino acids that comprised the TsaBgl L1 loop (Figure 5). Conversely, significantly diverse conformations were observed in the L2 and L3 loops, which comprised non-conserved amino acid residues and different amino acid numbers. MspBgl (PDB code: 4R27) and unbacBgl3 (6IER), which have a greater number of amino acids in their L2 loops than other Bgls, exhibited a helical structure in their corresponding L2 loops, and this was outwardly located with respect to the substrate-binding site (Figure 5). The L2 loops of HgrBgl (4MDO), OsaBgl (3GNO), OsaBgl2 (3PTK), OsaBgl3 (7D6A), OsaBgl4 (2RGL), ThaBgl (5JBO), HjeBgl (6KHT), and TreBgl (1CBG), which belong to the eukaryote class, were composed of more amino acids than the L2 loop of TsaBgl, resulting in a distinct conformation. The L2 loops of these Bgls were oriented in the opposite direction to the substrate binding site (Figure 5 and Supplementary Figure S5). These long L2 loops are considered to be involved in substrate accessibility or selectivity for bulk substrate molecules. Although the similarity between the amino acids constituting the different Bgl L2 loops was low, the conformation of the main TsaBgl L2 loop chain was almost the same as that of other Bgls having the same amino acid length. Thus, the conformational differences in the L2 loop between the different Bgls were considered to be based on the amino acid chain length. Not only were the amino acids in the L3 loop non-conserved, but they also exhibited the most significant conformational diversity in the loop region. No Bgl showed a similar L3 loop conformation to TsaBgl. In the model structures of AthBgl2, PpoBgl2, SspBgl, TmaBgl, and unbacBgl2, the L3 loop was partially built, indicating that the electron density maps of these molecules were disordered, with high flexibility. Unlike the L3 loop of the common Bgl, which lies around the substrate-binding site, the long L3 loop of ManBgl, comprising 45 amino acids, was found to cover its L2 and L4 loops. A unique substrate-binding pocket was formed by this distinct L3 loop conformation in the structure of ManBgl (Figure 5). This unique substrate-binding channel generated by the L3 loop exhibited a narrower substrate-binding pocket entrance in ManBgl than in other Bgls, potentially indicating that they have different substrate specificities.

Conversely, the structures of the L3 loops of SspBgl and unbacBgl2, which have a similar amino acid number to the L3 loop of ManBgl, were partially built, indicating that these Bgl L3 loops were flexible. More detailed studies are required to determine whether the ManBgl L3 loop forms a new substrate-accessible channel or whether it opens when the substrate approaches the substrate-binding pocket. The L4 loops of all Bgls, which showed highly conserved amino acid sequences, exhibited almost identical structural conformations.

Among the 45 previously reported crystal structures, AtuBgl, PpoBgl2, TmaBgl, PchBgl, OsaBgl, OsaBgl2, OsaBgl3, and OsaBgl4 were not only reported as native structures but also as structures with ligands bound to the active site (Table S3). To understand the structural changes in Bgl loops upon ligand binding, the loop conformations of native and ligand-bound Bgl structures were compared. It was observed that when a small ligand, such as glucose or a substrate-analog, bound to the Bgl active site, there was no significant change in the conformation of the main chain in all Bgl loops. However, comparing the native OsaBgl with octyl-β-d-tihio-glucoside-bound OsaBgl structures showed a conformational change in the L3 loop. The glucose moiety of octyl-beta-d-tihio-glucoside was bound to the active site of OsaBgl, while its aliphatic chain moiety laid around the substrate entrance. In native OsaBgl, the L3 loop, which was facing toward the substrate binding entrance, underwent a conformational change when bound to octyl-β-d-tihio-glucoside. However, there was no direct interaction between the aliphatic chain of octyl-β-d-tihio-glucoside and the OsaBgl L3 loop. In addition, the structure of glucose- or F-glucose-bound OsaBgl showed no change in the conformation of the L3 loop. In summary, no significant conformational changes were observed in the loops when a small-sized ligand was bound to Bgl.

## 3. Discussion

Understanding the structure of the four loops above the substrate-binding pocket of Bgl is important for elucidating the substrate access pathway and recognition of the enzyme. These loops show conformational diversity when compared to the crystal structures of other Bgl proteins [9], suggesting the occurrence of conformational changes in these loop regions during reactions. However, a detailed conformational analysis has not been conducted to confirm this speculation.

To better understand the conformation of the Bgl loops, we determined the high-resolution crystal structure of TsaBgl in two different crystal forms and compared the amino acid sequences and structures of proteins in the Bgl family. The crystal structures of the TsaBgl L1, L2, and L4 loops exhibited a rigid conformation via interaction with neighboring residues through hydrogen bonds and hydrophobic interactions. In contrast, the TsaBgl L3 loop showed relatively high flexibility and few interactions with neighboring residues. Although the N-terminal of the L3 loop exhibited two distinct conformations, these conformations did not affect the substrate-binding site pocket periphery. The relatively stable C-terminal shift of the L3 loop did not affect the size or surface charge of the substrate-binding site pocket. Although two distinct TsaBgl L3 loop conformations were observed in this study, we could not determine which conformation was preferred for substrate recognition. To obtain the conformational information on preferred loops for TsaBgl activation, further analysis of the crystal structure of TsaBgl complexed with a substrate should be performed using a mutant of the TsaBgl catalytic residue. In addition, to understand the function of the flexible N-terminal L3 loop, complex structural studies using bulk substrates need to be conducted. Furthermore, the L2 and L3 loops of Bgls differ in sequence and structure, and since they are involved in shaping the structure of the channel for substrate entry, they may be directly involved in determining the preferred substrates or activity of the enzyme.

Analysis of the amino acid sequence and structure of the loop region of the Bgl family showed that the structures of the L2 and L3 loop regions were very diverse. Particularly, different amino acid lengths in the Bgl L2 and L3 loops exhibited significant differences in conformation, which led to the differences in the entrance structure of the substrate-binding pocket in the Bgl family. Due to the significant differences in the amino acid sequences and structure of the Bgl L2 and L3 loops, the structural properties of the TsaBgl L2 and L3 loops cannot be applied to the structural changes and properties of other Bgl proteins. However, these differences in amino acid sequence and structure of the loop region at these substrate-binding entrances indicated that each Bgl may potentially use distinct substrate access pathways during substrate recognition. Furthermore, as the amino acid sequence of the L3 loop is non-conserved, the surface charge state around the Bgl substrate entrance varies depending on the amino acids comprising this loop. Meanwhile, the L1 and L4 loops show high similarity both in amino acid sequence and structure; therefore, the specific characteristics of each Bgl in the corresponding region could not be determined. Overall, the L1 and L4 loops above the Bgl substrate-binding pocket were structurally similar in conformation, while the L2 and L3 loops exhibited different conformations depending on the number and sequence of amino acids. Due to this diversity in the Bgl L2 and L3 loop regions in terms of the amino acid sequence and length, each Bgl protein exhibited unique properties. These unique L2 and L3 loop regions may result in differences in Bgl substrate specificity or selectivity since these loop regions are involved in substrate access or recognition. To verify substrate recognition and specificity in the L2 and L3 loops, further crystallographic and biochemical experiments will need to be conducted. Conversely, the structural analysis of TsaBgl showed that the L3 loop region could exhibit various conformations, and this can be a common structural feature in other Bgls, indicating relatively high flexibility. Thus, it can be speculated that the conformation of the L3 or L2 loop region in previously determined Bgl crystal structures may vary.

In this study, based on the Bgl crystal structure, we mainly assessed structural changes around the substrate entrance caused by structural changes in the loop. Previous mutagenesis studies have shown that loop mutations could potentially affect Bgl activity. For example, the activity of deletion mutants for the L3 loop was measured to assess the characteristics of the ManBgl loop [19]; the catalytic activities (kcat), substrate-binding ability (Km), Tm, and optimal temperatures of ManBgl-ΔL3 and ManBgl were 0.46 × 10^3^ s^−1^ and 6.7 × 10^3^ s^−1^, 19.39 mmol L^−1^ and 5.9 mmol L^−1^, <25 and 36.5 °C, and 20 and 25 °C, respectively [19]. These results indicate that shortening the ManBgl L3 loop induces a significant decrease in catalytic activity, substrate binding ability, thermostability, and optimal temperature. This indicates that enzyme activity characteristics may change depending on the mutation of the loop region in the Bgl family.

In summary, we reported the structural characteristics of the TsaBgl substrate entrance loop and observed changes in the size of the substrate access pocket or the surface charge depending on the conformation of the L3 loop. By comparing the amino acid sequences and structures of the loops of various Bgls, the amino acid sequences and structural specificity of the Bgl L2 and L3 loops were determined. Although this study clarified the structural diversity of the Bgl loops, it did not determine the involvement of structural differences in these Bgl loops in substrate recognition and enzyme activity. Thus, further research is needed to determine whether these loops are functionally involved in substrate recognition at various conformations, as well as in the start (ready for substrate binding) or end (product release) of the catalytic cycle in the catalytic mechanism of glucosidase. Furthermore, it will be necessary to analyze the structure and function of various loops through mutagenesis studies and determine the crystal structures of the substrate-bound and non-substrate-bound states of Bgl. These studies will provide useful insights into engineering approaches for Bgl to enhance its enzyme activity for future bioenergy production. In conclusion, these findings expand our knowledge on the structural properties of Bgl proteins and provide a structure-based functional analysis of TsaBgl.

## 4. Materials and Methods

### 4.1. Sample Preparation

Cloning and protein preparation were performed following methods detailed in a previous study [18]. Briefly, codon-optimized TsaBgl (UniProt: I3VXG7) was synthesized and cloned into the pBT7 vector (Bioneer, Daejeon, Republic of Korea). *Escherichia coli* BL21 (DE3) containing the recombinant DNA was cultured in LB medium supplemented with 50 mg/mL ampicillin at 37 °C. When the OD_600_ reached 0.4–0.8, recombinant protein expression was induced via the addition of 0.5 mM isopropyl-d-1-thiogalactopyranoside (IPTG). Then, the cells were incubated in a shaking incubator at 18 °C for 18 h. Harvested cells were placed in a lysis buffer containing 50 mM Tris-HCl, pH 8.0, 200 mM NaCl, and 20 mM imidazole and disrupted on ice using a sonicator. Cell debris was removed via centrifugation at 18,894 g for 30 min. The supernatant was loaded onto a column containing Ni-NTA resin. The column was washed with a buffer containing 50 mM Tris-HCl, pH 8.0, 200 mM NaCl, and 20 mM imidazole. Proteins were eluted using a buffer containing 50 mM Tris-HCl, pH 8.0, 200 mM NaCl, and 300 mM imidazole. The N-terminal hexahistidine-tag was removed via incubation with thrombin (Sigma Aldrich, St. Louis, MO, USA) overnight at 20 °C. Proteins were concentrated using a Centricon filter (Merck Millipore, Burlington, MA, USA; cut-off: 30 kDa) and loaded onto a Sephacryl-100 column (GE Healthcare, Chicago, IL, USA) with buffer containing 10 mM Tris-HCl, pH 8.0, and 200 mM NaCl. Protein purity was determined through SDS-PAGE using Coomassie Brilliant Blue. Protein concentration was measured using the UV-Vis spectroscopy NanoDrop, a NanoDrop 1000 spectrophotometer (Thermo Fisher Scientific, Waltham, MA, USA). The proteins were concentrated to 20 mg/mL using a Centricon (10 kDa cut-off, Millipore Merck, Burlington, MA, USA).

### 4.2. Crystallization

Protein crystallization was performed using the sitting-drop vapor diffusion method at 20 °C. The TsaBgl solution (20 mg/mL, 0.5 μL) was mixed with a reservoir solution (0.5 μL) obtained from a commercially available crystallization screen kit. TsaBgl microcrystals were obtained using a solution containing 0.1 M Tris-HCl, pH 7.5, 30% (*w*/*v*), and 0.2 M MgCl_2_. The crystal optimization experiment was performed using a 24-well VDX plate (Hampton Research, Aliso Viejo, CA, USA). Suitable TsaBgl crystals for X-ray diffraction analysis were obtained from a reservoir solution containing 0.1 M Tris-HCl, pH 7.5–8.0, 15–20% (*w*/*v*) PEG 4000, and 0.2 M MgCl_2_, equilibrated with the reservoir solution (500 μL), and incubated at 20 °C. The protein crystals appeared within two weeks.

### 4.3. X-ray Diffraction Data Collection

Diffraction data were collected at Beamline 11C at the Pohang Light Source II (PLS-II, Pohang, Republic of Korea). TsaBgl crystals were cryoprotected using a reservoir solution supplemented with 20% (*v*/*v*) ethylene glycol. X-ray energy was set at 12.569 keV. Diffraction data were collected at a cryogenic temperature of 100 K. Diffraction images were recorded on a Pilatus3 6M detector. X-ray diffraction data were indexed, integrated, and scaled using the HKL2000 program [20].

### 4.4. Structure Determination

The phasing problems were solved with the molecular replacement method using the MOLREP (version 11.2.08) program [21]. The TsaBgl (PDB ID: 7E5J) crystal structure [18] was used as the search model. The model structure was built using the Coot (version 0.9.6) program [22]. Structure refinement was performed using the refine.phenix function in the PHENIX (version 1.20.1-4487) program [23]. The geometry of the final model was validated using MolProbity (version 4.5.1) [24]. The protein structure was visualized using PyMOL (version 2.4.1.) (http://pymol.org).

### 4.5. Analysis of the Bgl Loop Region

The amino acid sequences and structures of 46 Bgls were obtained from UniProt (https://www.uniprot.org, access date: 9 August 2023) and the Protein Data Bank (https://www.rcsb.org, access date: 9 August 2023), respectively. Amino acid sequence alignment was performed using Clustal Omega [25]. Structure-based sequence alignment was visualized using ESPript 3 (version 3.0.10) [26].

## Figures and Tables

**Table 1:** Data collection and refinement statistics.

Data	Data I	Data II	Data III	Data IV
Wavelength (Å)	0.9864	0.9864	0.9864	0.9864
Space group	P1	P1	P2_1_2_1_2_1_	P2_1_2_1_2_1_
Unit cell (Å)				
a	63.414	63.213	64.970	65.066
b	72.831	72.739	70.948	71.211
c	97.490	97.385	98.876	99.151
α	92.495	92.484	90.000	90.000
β	81.281	91.431	90.000	90.000
γ	95.128	95.215	90.000	90.000
Molecule/asym.	4	4	1	1
Resolution (Å) ^a^	50.00–1.90 (1.93–1.90)	50.00–2.10 (2.14–2.10)	50.00–1.50 (1.53–1.50)	50.00–1.60 (1.63–1.60)
Unique reflections	123,062 (6212)	90,740 (4342)	71,920 (3026)	60,041 (2923)
Completeness (%)	90.7 (91.5)	90.9 (87.2)	97.4 (83.5)	98.8 (97.8)
Redundancy	3.7 (3.4)	3.7 (3.4)	4.1 (2.4)	5.7 (4.6)
Mean *I*/σ(*I*)	10.78 (1.90)	9.02 (1.92)	15.41 (2.47)	14.63 (2.15)
CC1/2	0.974 (0.563)	0.977 (0.706)	0.973 (0.771)	0.981 (0.585)
CC*	0.993 (0.849)	0.994 (0.910)	0.993 (0.933)	0.995 (0.859)
**Refinement**				
Resolution (Å)	49.89–1.90	49.81–2.10	49.44–1.50	49.58–1.61
R_work_	0.1694	0.1599	0.1546	0.1506
R_free_	0.1952	0.2019	0.1712	0.1762
RMS deviations				
Bonds (Å)	0.003	0.004	0.003	0.009
Angles (degree)	0.632	0.670	0.648	0.963
*B* factor (Å^2^)				
Protein	21.99	23.91	12.28	13.40
Water	31.42	31.42	27.10	27.97
Ramachandran plot (%)				
Most favored	96.99	96.48	97.74	97.96
Allowed	2.90	3.46	2.26	2.04
Outliers	0.11	0.06	0.0	0.0
PDB code	8WFT	8WFU	8WFV	8WFW

^a^ Values in parentheses are for outer shells.

## Data Availability

The diffraction images of TsaBgl have been deposited in the Zenodo (https://doi.org/10.5281/zenodo.8424379). The coordinates and structure factors for the protein crystals in this study are available in the Protein Data Bank (https://www.rcsb.org/) under entries 8WFT (P1, data I), 8WFU (P1, data II), 8WFV (P2_1_2_1_2_1_, Data III), and 8WFW (P2_1_2_1_2_1_, Data IV).

## Supplementary Materials

The following supporting information can be downloaded at: https://www.mdpi.com/article/10.3390/molecules28237807/s1. Figure S1: Electron density map of L3 loops of TsaBgl; Figure S2: B-factor putty representation of TsaBgl molecules; Figure S3: Phylogenic tree of Bgl proteins; Figure S4: Structure-based sequence alignment of Bgl proteins; Figure S5: Structural comparison of the loop structures of Bgl proteins; Figure S6: Superimposition of native and ligand-bound Bgl proteins; Table S1: Analysis of temperature factor of TsaBgl; Table S2: Sequence information of Bgl used in this study; Table S3: Crystal structures of ligand-bound Bgl proteins. References [25,27] are cited in the supplementary materials.
