# Supplementary material for: The Conformational Change of the L3 Loop Affects the Structural Changes in the Substrate Binding Pocket Entrance of β-Glucosidase
Source: Molecules. 2023 Nov 27;28(23):7807. doi: 10.3390/molecules28237807 (PMC10707726; doi:10.3390/molecules28237807)
Supplement: Supplementary file 1 [file molecules-28-07807-s001.zip › molecules-2660406-supplementary.pdf]

Supplementary Data

The conformational change of the L3 loop affects the structural changes in the substrate binding pocket entrance of β-glucosidase

Ki Hyun Nam*

College of General Education, Kookmin University, Seoul 02707, Republic of Korea

* Correspondence: structure@kookmin.ac.kr

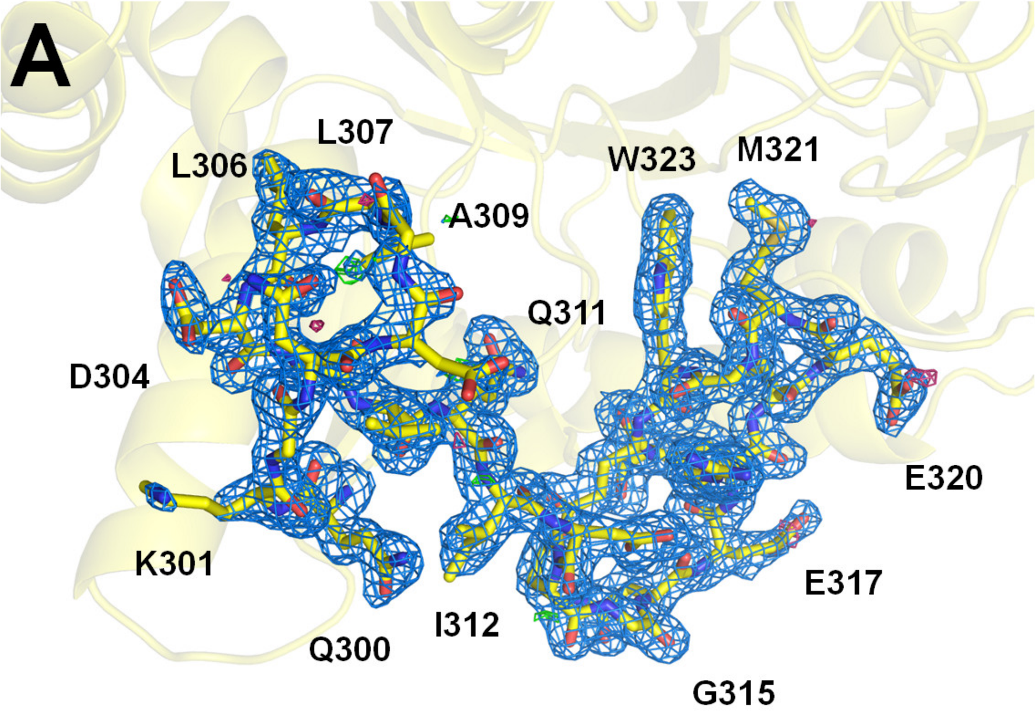

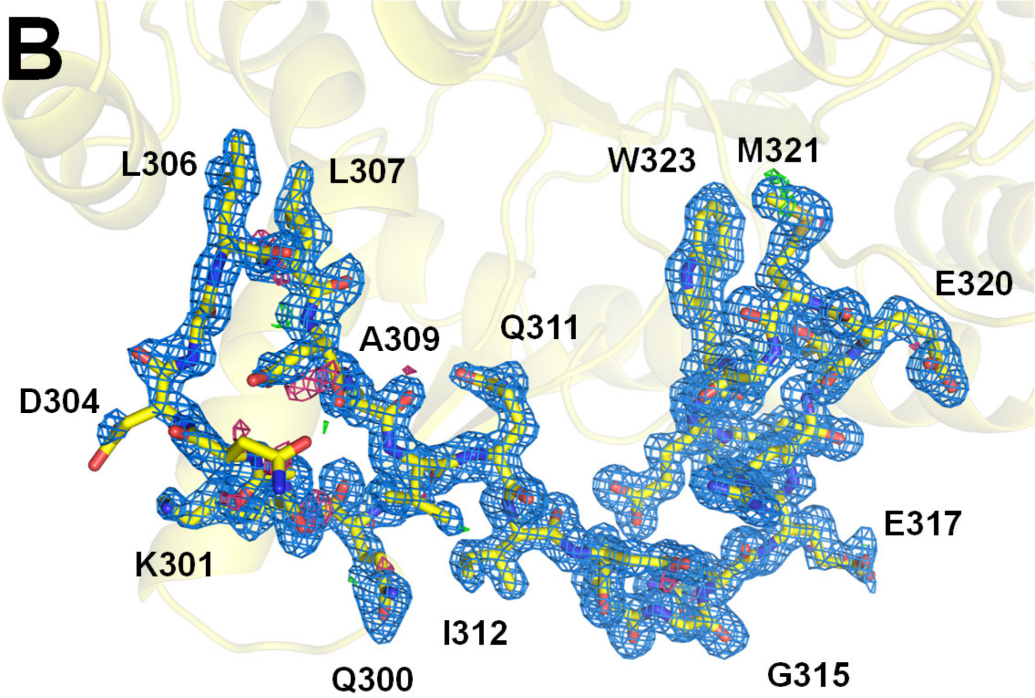

Supplementary Figure S1. 2mFo-Fc (1σ, blue mesh) and Fo-Fc (3σ, green mesh; -3σ, red mesh) electron density map of (A) folded L3 loop (Data I, chain A) and (B) straight L3 loop (Data III, chain A) of TsaBgl.

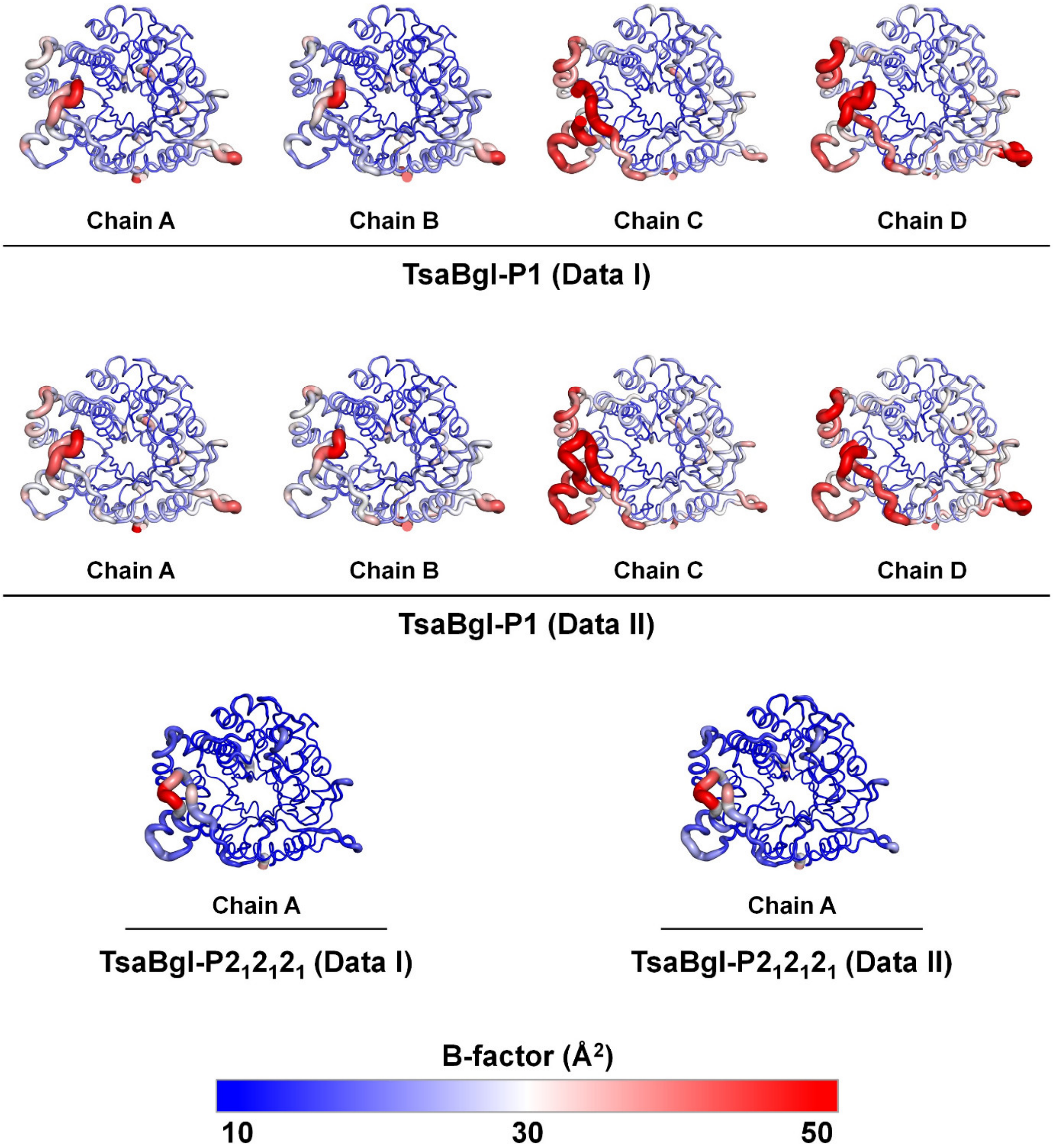

Supplementary Figure S2. B-factor putty representation of TsaBgl molecules from the two crystals form datasets, P1 and P2$_1$2$_1$2$_1$.

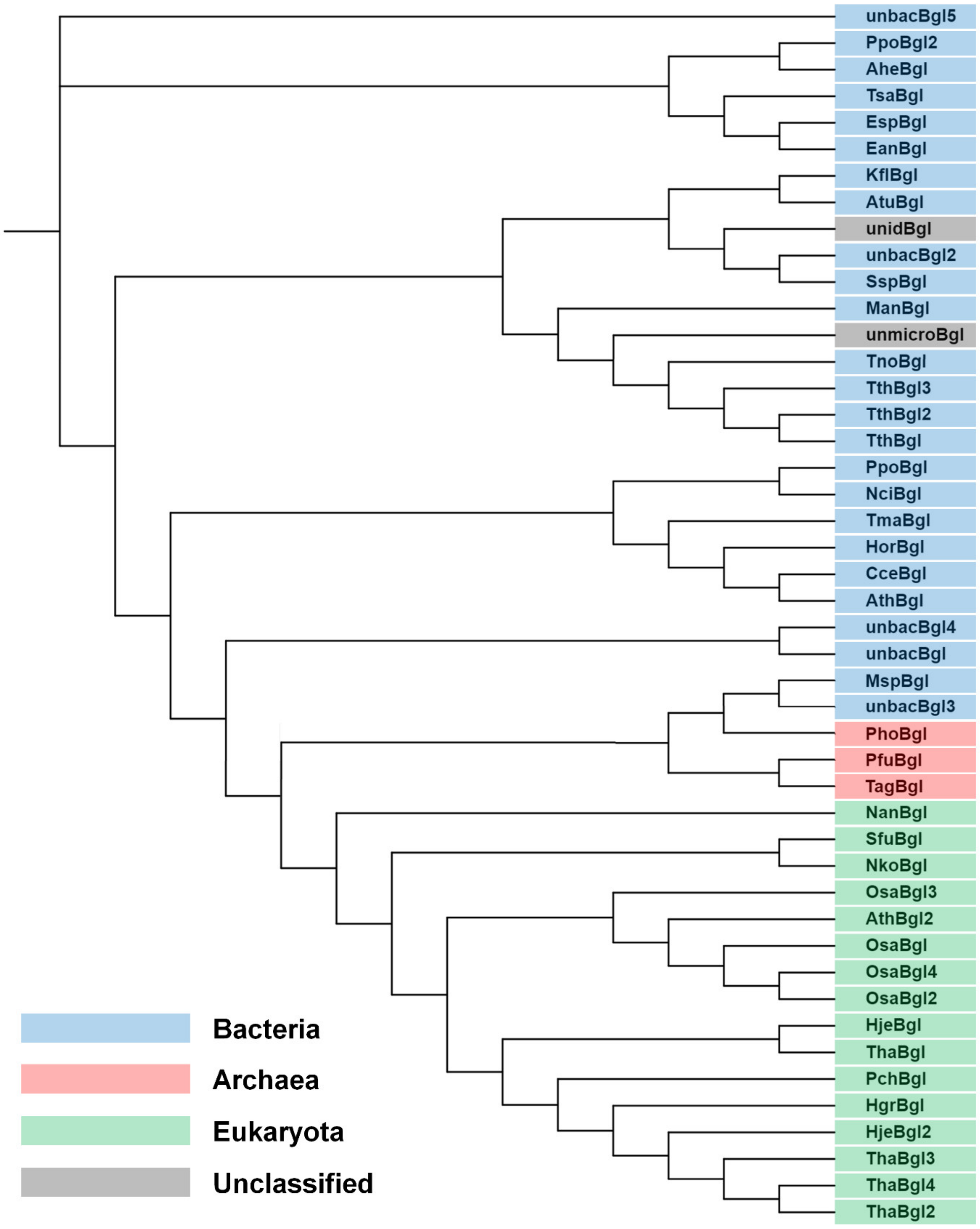

Supplementary Figure S3. Phylogenic tree of Bgl protein from *Thermoanaerobacterium saccharolyticum* (TsaBgl, I3VXG7), *Pyrococcus furiosus* (PfuBgl, E7FHY4), *Pyrococcus horikoshii* (PhoBgl, O58104), *Thermosphaera aggregans* (TagBgl, Q9YGA8), Acetivibrio thermocellus (AthBgl, P26208), *Agrobacterium tumefaciens* (AtuBgl, A0A2I4PGZ0), *Alicyclobacillus herbarius* (AheBgl, A0A8I3B065), *Clostridium cellulovorans* (CceBgl, Q53EH2), *Exiguobacterium antarcticum* (EanBgl, K0A8J9), *Exiguobacterium* sp. (EspBgl,

C4L1S4), *Halothermothrix orenii* (HorBgl, B8CYA8), *Kribbella flavida* (KflBgl, D2PL27), *Microbacterium* sp.(MspBgl, L0ELG0), *Micrococcus antarcticus* (ManBgl, B9V8P5), *Niallia circulans* (NciBgl, Q03506), *Paenibacillus polymyxa* (PpoBgl, P22073), *Paenibacillus polymyxa* (PpoBgl2, P22505), *Streptomyces* sp. (SspBgl, Q59976), *Thermotoga maritima* (TmaBgl, Q08638), *Thermus nonproteolyticus* (TnoBgl, Q9L794), *Thermus thermophilus* (TthBgl, Q53W75), *Thermus thermophilus* (TthBgl2, Q8GEB3), *Thermus thermophiles* (TthBgl3, Q9RA61), uncultured bacterium (unbacBgl, A0A0F7KKB7), uncultured bacterium (unbacBgl2, A0A1L3HS62), uncultured bacterium (unbacBgl3, A0A4D6T7S3), uncultured bacterium (unbacBgl4, A0A5B9BHU3), uncultured bacterium (unbacBgl5, Q0GMU3),Arabidopsis thaliana (AthBgl2, A0A654G6E3), *Humicola grisea* var. thermoidea (HgrBgl, O93784), *Nannochloris* (NanBgl, A0A452CSM4), *Neotermes koshunensis* (NkoBgl, Q8T0W7), *Oryza sativa* subsp. japonica (OsaBgl, Q8L7J2), *Oryza sativa* subsp. indica (OsaBgl2, B8AVF0), *Oryza sativa* subsp. japonica (OsaBgl3, Q7XSK0), *Oryza sativa* subsp. japonica (OsaBgl4, Q75I93), *Phanerodontia chrysosporium* (PchBgl, Q25BW5), *Spodoptera frugiperda* (SfrBgl, O61594), *Trichoderma harzianum* (ThaBgl, A0A0F9XM91), *Trichoderma harzianum* (ThaBgl2, A0A0F9ZQA8), *Trichoderma harzianum* (ThaBgl3, A3FPG4), *Trichoderma harzianum* (ThaBgl4, A0A2T4AR08), *Hypocrea jecorina* (HjeBgl, G0RIF5), *Hypocrea jecorina* (HjeBgl2, O93785), *Trifolium repens* (TreBgl, P26205), uncultured microorganism (unmicroBgl, A0A1E1FFN6), unidentified (unidBgl, A0A2I2LGB3). Phylogenetic tree data was generated with Clustal Omga [1] and visualized with Interactive Tree Of Life (iTOL) v5 [2].

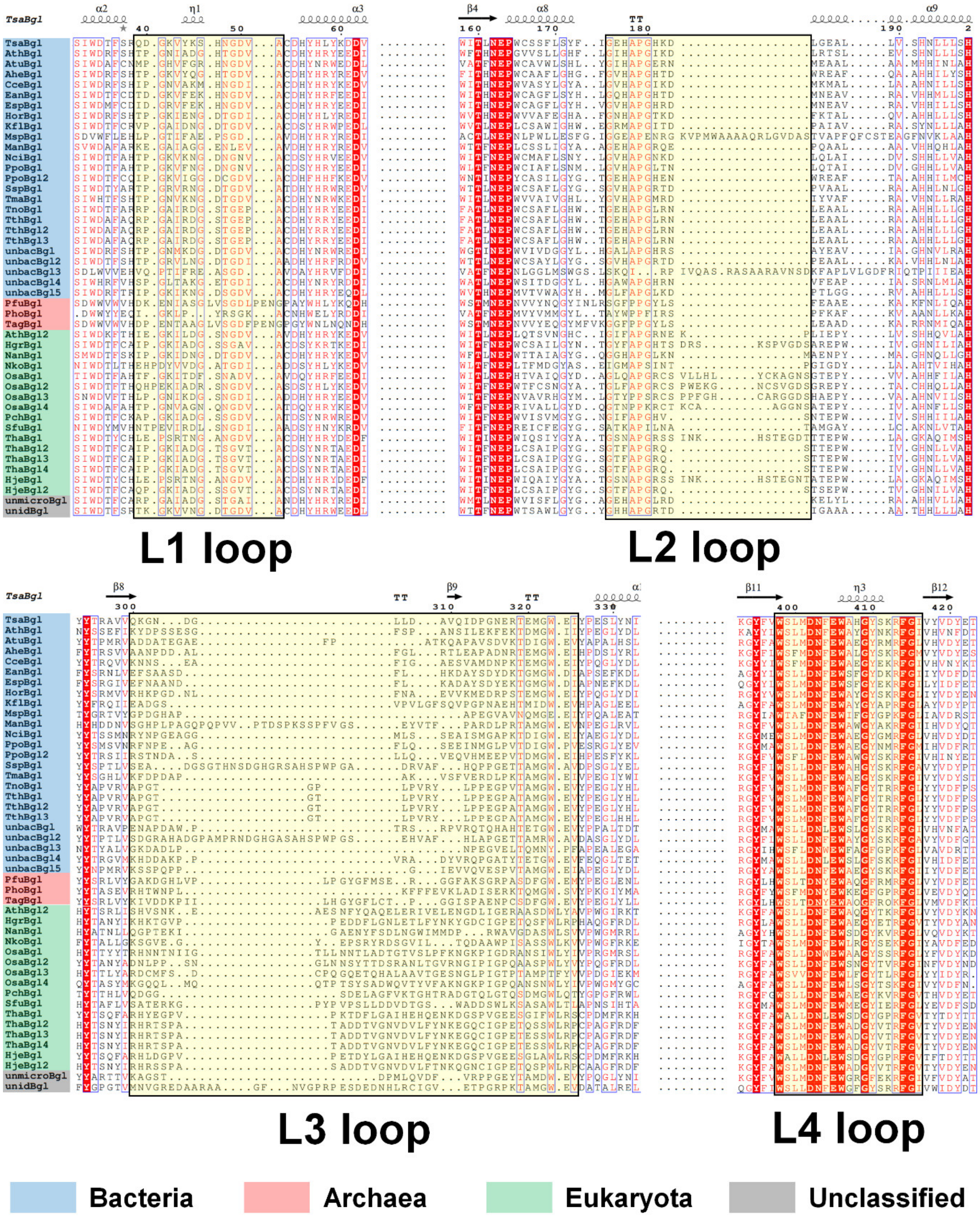

Supplementary Figure S4. Structure-based sequence alignment of β-glucosidase from *Thermoanaerobacterium saccharolyticum* (TsaBgl, UniProt code: I3VXG7), *Pyrococcus furiosus* (PfuBgl, E7FHY4), *Pyrococcus horikoshii* (PhoBgl, O58104), *Thermosphaera aggregans* (TagBgl, Q9YGA8), *Acetivibrio thermocellus* (AthBgl, P26208), *Agrobacterium tumefaciens* (AtuBgl, A0A2I4PGZ0), *Alicyclobacillus herbarius* (AheBgl, A0A8I3B065), *Clostridium cellulovorans* (CceBgl, Q53EH2), *Exiguobacterium antarcticum* (EanBgl, K0A8J9), *Exiguobacterium* sp. (EspBgl, C4L1S4), *Halothermothrix orenii* (HorBgl, B8CYA8), *Kribbella*

flavida (KflBgl, D2PL27), *Microbacterium* sp.(MspBgl, L0ELG0), *Micrococcus antarcticus* (ManBgl, B9V8P5), *Niallia circulans* (NciBgl, Q03506), *Paenibacillus polymyxa* (PpoBgl, P22073), *Paenibacillus polymyxa* (PpoBgl2, P22505), *Streptomyces* sp. (SspBgl, Q59976), *Thermotoga maritima* (TmaBgl, Q08638), *Thermus nonproteolyticus* (TnoBgl, Q9L794), *Thermus thermophilus* (TthBgl, Q53W75), *Thermus thermophilus* (TthBgl2, Q8GEB3), *Thermus thermophiles* (TthBgl3, Q9RA61), uncultured bacterium (unbacBgl, A0A0F7KKB7), uncultured bacterium (unbacBgl2, A0A1L3HS62), uncultured bacterium (unbacBgl3, A0A4D6T7S3), uncultured bacterium (unbacBgl4, A0A5B9BHU3), uncultured bacterium (unbacBgl5, Q0GMU3), *Arabidopsis thaliana* (AthBgl2, A0A654G6E3), *Humicola grisea* var. thermoidea (HgrBgl, O93784), *Nannochloris* (NanBgl, A0A452CSM4), *Neotermes koshunensis* (NkoBgl, Q8T0W7), *Oryza sativa* subsp. japonica (OsaBgl, Q8L7J2),Oryza sativa subsp. indica (OsaBgl2, B8AVF0), *Oryza sativa* subsp. japonica (OsaBgl3, Q7XSK0), *Oryza sativa* subsp. japonica (OsaBgl4, Q75I93), *Phanerodontia chrysosporium* (PchBgl, Q25BW5), *Spodoptera frugiperda* (SfrBgl, O61594), *Trichoderma harzianum* (ThaBgl, A0A0F9XM91), *Trichoderma harzianum* (ThaBgl2, A0A0F9ZQA8), *Trichoderma harzianum* (ThaBgl3, A3FPG4), *Trichoderma harzianum* (ThaBgl4, A0A2T4AR08), *Hypocrea jecorina* (HjeBgl, G0RIF5), *Hypocrea jecorina* (HjeBgl2, O93785), uncultured microorganism (unmicroBgl, A0A1E1FFN6), and unidentified (unidBgl, A0A2I2LGB3).

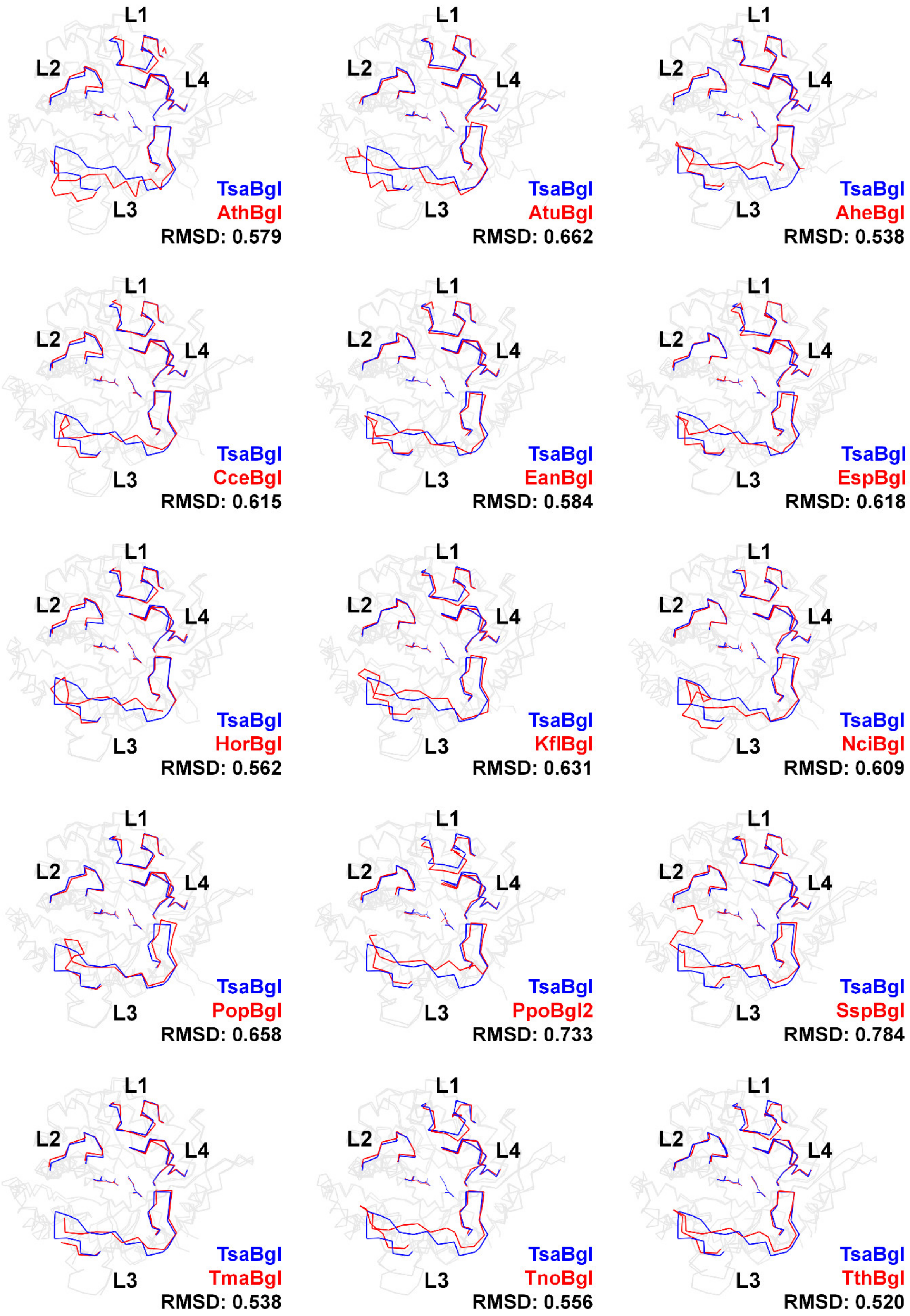
L1
L2
L4
L3
TsaBgl
AthBgl
RMSD: 0.579
L1
L2
L4
L3
TsaBgl
AtuBgl
RMSD: 0.662
L1
L2
L4
L3
TsaBgl
AheBgl
RMSD: 0.538
L1
L2
L4
L3
TsaBgl
CceBgl
RMSD: 0.615
L1
L2
L4
L3
TsaBgl
EanBgl
RMSD: 0.584
L1
L2
L4
L3
TsaBgl
EspBgl
RMSD: 0.618
L1
L2
L4
L3
TsaBgl
HorBgl
RMSD: 0.562
L1
L2
L4
L3
TsaBgl
KflBgl
RMSD: 0.631
L1
L2
L4
L3
TsaBgl
NciBgl
RMSD: 0.609
L1
L2
L4
L3
TsaBgl
PopBgl
RMSD: 0.658
L1
L2
L4
L3
TsaBgl
PpoBgl2
RMSD: 0.733
L1
L2
L4
L3
TsaBgl
SspBgl
RMSD: 0.784
L1
L2
L4
L3
TsaBgl
TmaBgl
RMSD: 0.538
L1
L2
L4
L3
TsaBgl
TnoBgl
RMSD: 0.556
L1
L2
L4
L3
TsaBgl
TthBgl
RMSD: 0.520

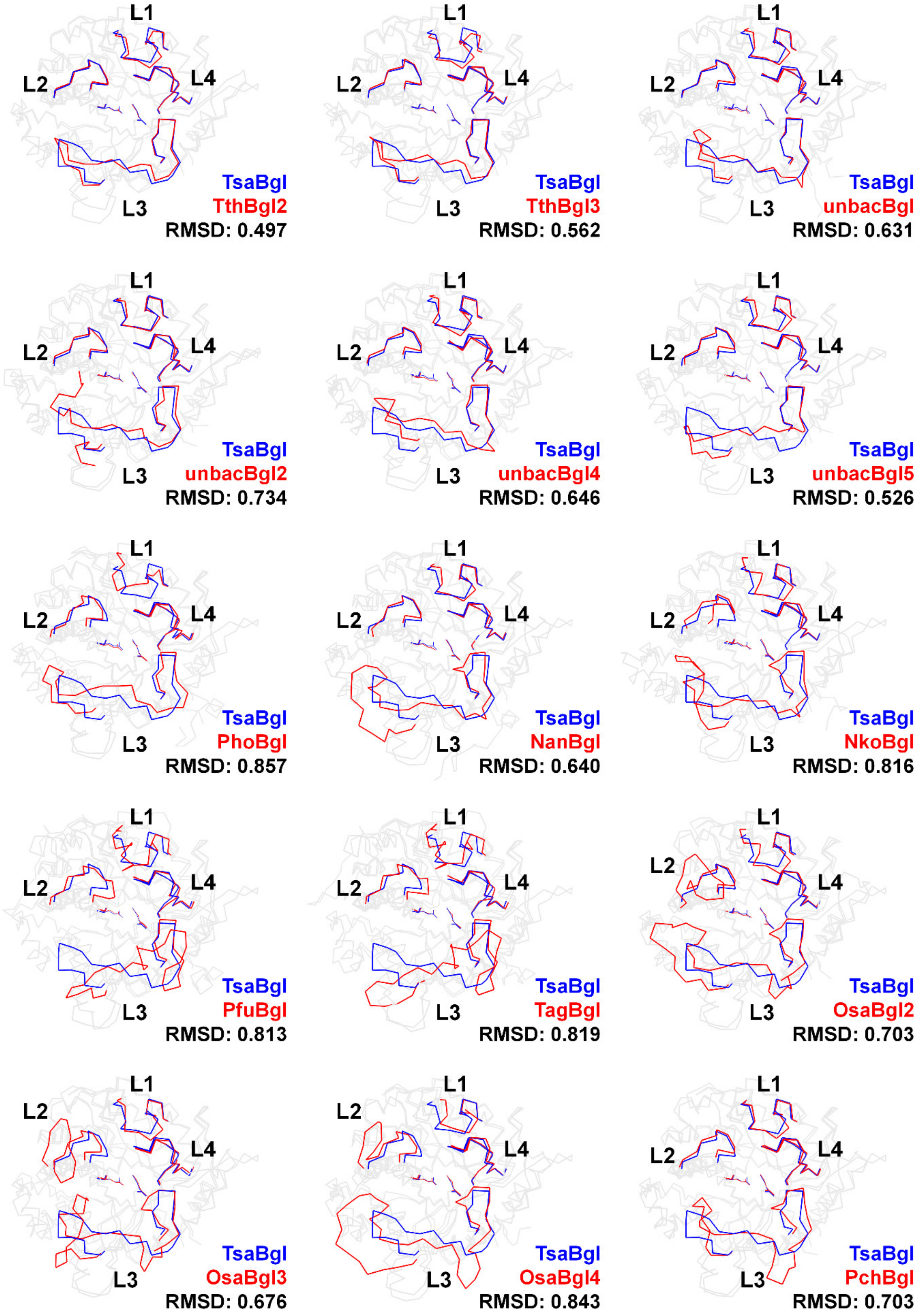
L1
L2
L4
L3
TsaBgl
TthBgl2
RMSD: 0.497
TsaBgl
TthBgl3
RMSD: 0.562
TsaBgl
unbacBgl
RMSD: 0.631
TsaBgl
unbacBgl2
RMSD: 0.734
TsaBgl
unbacBgl4
RMSD: 0.646
TsaBgl
unbacBgl5
RMSD: 0.526
TsaBgl
PhoBgl
RMSD: 0.857
TsaBgl
NanBgl
RMSD: 0.640
TsaBgl
NkoBgl
RMSD: 0.816
TsaBgl
PfuBgl
RMSD: 0.813
TsaBgl
TagBgl
RMSD: 0.819
TsaBgl
OsaBgl2
RMSD: 0.703
TsaBgl
OsaBgl3
RMSD: 0.676
TsaBgl
OsaBgl4
RMSD: 0.843
TsaBgl
PchBgl
RMSD: 0.703

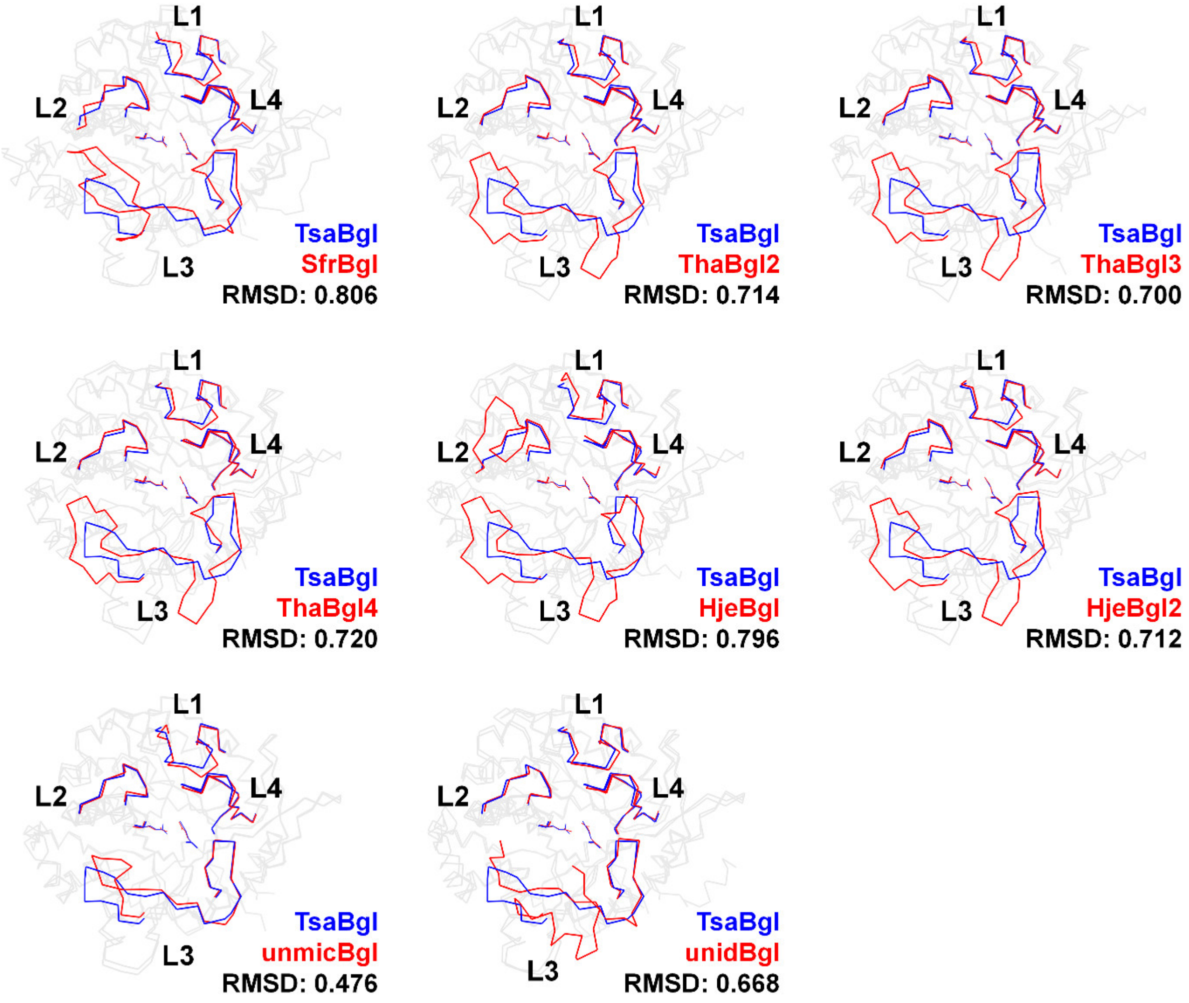

Supplementary Figure S5. Structural comparison of the loop structures on TsaBgl substrate binding pockets with TagBgl (PDB code: 1QVB), AthBgl (5OGZ), AtuBgl (6RJK), AheBgl (6YN7), CceBgl (3AHX), EanBgl(5DT5), EspBgl (6WIU), HorBgl (3TA9), KflBgl (6M6L), NciBgl (1QOX), PpoBgl (1BGA), PpoBgl2 (2JIE), SspBgl (1GNX), TmaBgl (1OD0), TnoBgl (1NP2), TthBgl (1UG6), TthBgl2 (4BCE), TthBgl3 (3ZJK), unbacBgl (5GNX), unbacBgl2 (7BBS), unbacBgl4 (6JFP), unbacBgl5 (3CMJ), PhoBgl (1VFF), NanBgl (5YJ7), NkoBgl (3AHZ), PfuBgl (3APG), TsaBgl (7E5J), OsaBgl2 (3PTK), OsaBgl3 (7D6A), OsaBgl4 (2RGL), PchBgl (2E3Z), SfrBgl (5CG0), ThaBgl2 (5JBK), ThaBgl3 (5BWF), ThaBgl4 (6EFU), HjeBgl (6KHT), HjeBgl2 (3AHY), unmicBgl (5XGZ) and unidBgl (5WKA).

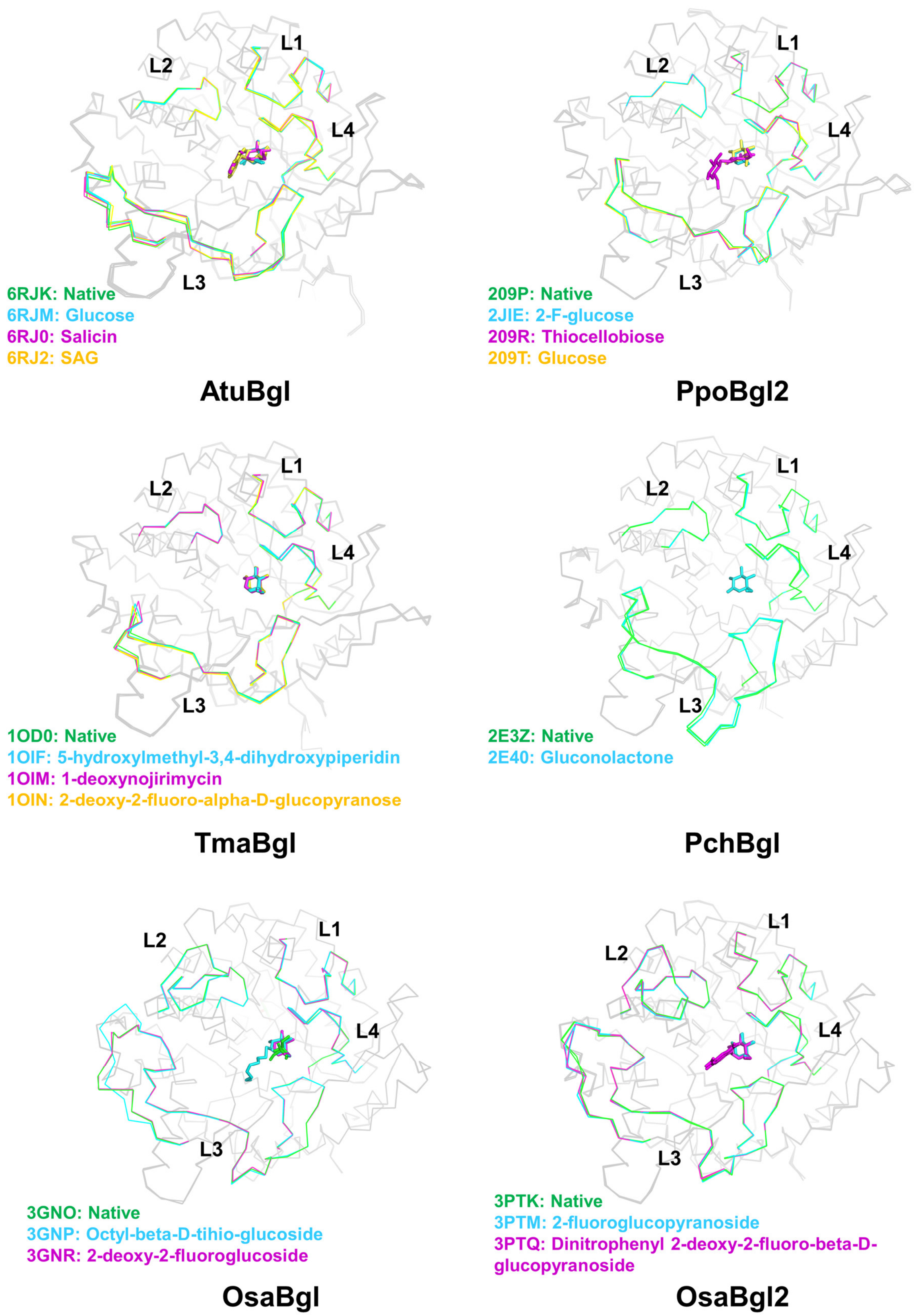
L1
L2
L3
L4
6RJK: Native
6RJM: Glucose
6RJ0: Salicin
6RJ2: SAG
AtuBgl
L1
L2
L3
L4
209P: Native
2JIE: 2-F-glucose
209R: Thiocellobiose
209T: Glucose
PpoBgl2
L1
L2
L3
L4
1OD0: Native
1OIF: 5-hydroxylmethyl-3,4-dihydroxypiperidin
1OIM: 1-deoxynojirimycin
1OIN: 2-deoxy-2-fluoro-alpha-D-glucopyranose
TmaBgl
L1
L2
L3
L4
2E3Z: Native
2E40: Gluconolactone
PchBgl
L1
L2
L3
L4
3GNO: Native
3GNP: Octyl-beta-D-tihio-glucoside
3GNR: 2-deoxy-2-fluoroglucoside
OsaBgl
L1
L2
L3
L4
3PTK: Native
3PTM: 2-fluoroglucopyranoside
3PTQ: Dinitrophenyl 2-deoxy-2-fluoro-beta-D-glucopyranoside
OsaBgl2

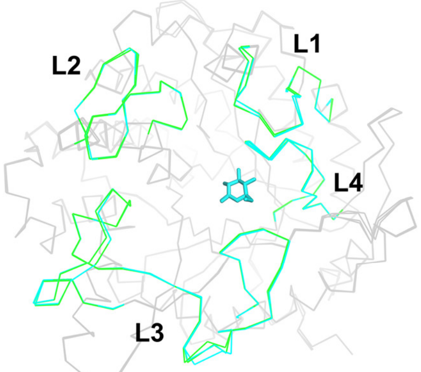

OsaBgl3

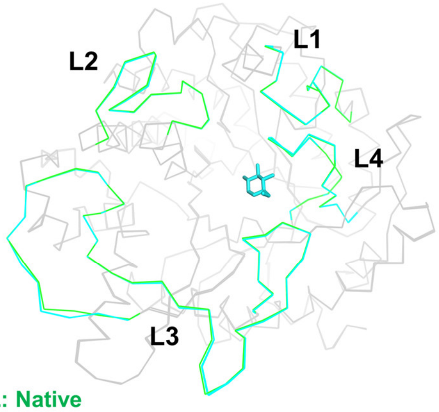

OsaBgl4

Supplementary Figure S6. Superimposition of native and ligand-bound Bgl for AtuBgl, ProBgl2, TmaBgl, PchBgl, OsaBgl, OsaBgl2, OsaBgl3, and OsaBgl4. The PDB codes and Bgl bound ligand were shown superimposition structures. The detailed information of the Bgl structures were shown in Table S3.

Table S1. Analysis of temperature factor (Å^2) of TsaBgl and its four loops.

Data	Chain	L1	L2	L3	L4	Whole
Data I	A	17.31	19.26	27.64	15.36	20.94
	B	15.79	17.94	26.16	14.64	20.27
	C	22.62	24.01	37.74	17.23	22.73
	D	25.81	25.83	38.91	19.92	24.01
Data I	A	19.79	21.06	30.04	17.31	22.91
	B	17.55	18.55	28.79	16.37	22.19
	C	25.74	25.01	43.46	19.89	24.97
	D	27.55	27.43	39.42	21.48	25.56
Data III	A	12.77	13.66	21.13	8.77	13.42
Data IV	A	13.82	14.31	23.47	9.40	12.30

Table S2. Sequence information of Bgl used in this study.

**Archaea**				
	**Protein Name**	**Organism**	**UniProt**	**PDB**
PfuBgl	β-glucosidase / β-rutinosidase	*Pyrococcus furiosus*	Q51723	3APG, 3WDP, 3WQ8
PhoBgl	alkyl β-glucosidase	*Pyrococcus horikoshii* OT3	O58104	1VFF
TagBgl	β-glucosidase	*Thermosphaera aggregans* M11TL	Q9YGA8	1QVB
**Bacteria**				
	**Protein Name**	**Organism**	**UniProt**	**PDB**
AthBgl	exo-β-glucosidase 1A	*Acetivibrio thermocellus* ATCC 27405	P26208	5OGZ
AtuBgl	SghA	*Agrobacterium tumefaciens A6*	A0A2I4PGZ0	6RJK, 6RJM, 6RJO, 6RK2
AheBgl	β-glucosidase	*Alicyclobacillus herbarius*	A0A8I3B065	6YN7
CceBgl	β-glucosidase A	*Clostridium cellulovorans*	Q53EH2	3AHX
EanBgl	β-glucosidase	*Exiguobacterium antarcticum* B7 Eab7	K0A8J9	5DT5, 5DT7
EspBgl	EAT1b_2183	*Exiguobacterium* sp. AT1b	C4L1S4	6WIU
HorBgl	β-glucosidase A	*Halothermothrix orenii H* 168	B8CYA8	3TA9, 4PTV, 4PTW, 4PTX
KflBgl	β-glucosidase	*Kribbella flavida* DSM 17836	D2PL27	6M6L, 6M6M
MspBgl	β-glycosidase	*Microbacterium* sp. Gsoil167	L0ELG0	4R27
ManBgl	β-glucosidase	*Micrococcus antarcticus*	B9V8P5	3W53
NciBgl	β-glucosidase	*Niallia circulans subsp. alkalophilus*	Q03506	1QOX
PpoBgl	β-glucosidase A	*Paenibacillus polymyxa*	P22073	1BGA, 1BGG, 1E4I, 1TR1, 1UYQ, 6QWI, 6R4K
PpoBgl2	β-glucosidase B	*Paenibacillus polymyxa*	P22505	2JIE, 2O9P, 2O9R, 2O9T, 2Z1S
SspBgl	β-glucosidase	*Streptomyces* sp. QM-B814	Q59976	1GNX, 1GON
TsaBgl	β-glucosidase	*Thermoanaerobacterium saccharolyticum* JW/SL-YS485	I3VXG7	7E5J
TmaBgl	β-glucosidase A	*Thermotoga maritima* MSB8	Q08638	1OD0, 1OIF, 1OIM, 1OIN, 1UZ1, 1W3J, 2CBU, 2CBV, 2CES, 2CET, 2J75, 2J77, 2J78, 2J79, 2J7B,

				2J7C, 2J7D, 2J7E, 2J7F, 2J7G, 2J7H, 2JAL, 2VRJ, 2WBG, 2WC3, 2WC4, 5N6S, 5N6T, 5OSS,
TnoBgl	β-glycosidase	*Thermus nonproteolyticus* HG102	Q9L794	1NP2
TthBgl	β-glycosidase / β-glucosidase	*Thermus thermophilus* HB8	Q53W75	1UG6
TthBgl2	β-glycosidase / β-glucosidase	*Thermus thermophilus*	Q8GEB3	4BCE
TthBgl3	β-glycosidase	*Thermus thermophilus* TH125	Q9RA61	3ZJK
unbacBgl	β-glucosidase	uncultured bacterium	A0A0F7KKB7	5GNX, 5GNY, 5GNZ, 7BBS
unbacBgl2	β-glucosidase Bg10	uncultured bacterium	A0A1L3HS62	7BBS
unbacBgl3	β-glucosidase	uncultured bacterium	A0A4D6T7S3	6IER
unbacBgl4	β-glucosidase Bgl15	uncultured bacterium	A0A5B9BHU3	6JFP
unbacBgl5	β-glucosidase / β-glycosidase	uncultured bacterium	Q0GMU3	3CMJ, 3FIY, 3FIZ, 3FJ0, 4HZ6, 4HZ7, 4HZ8
**Eukaryota**				
	**Protein Name**	**Organism**	**UniProt**	**PDB**
AthBgl2	β-glucosidase	*Arabidopsis thaliana*	A0A654G6E3	7F3A
HgrBgl	β-glucosidase	*Humicola grisea var. thermoidea* IFO9854 / *Humicola insolens* RP86	O93784	4MDO, 4MDP
NanBgl	β-glucosidase BGLN1	*Nannochloris*	A0A452CSM4	5YJ7
NkoBgl	β-glucosidase	*Neotermes koshunensis*	Q8T0W7	3AHZ, 3AI0, 3VIF, 3VIG, 3VIH, 3VII, 3VIJ, 3VIK, 3VIL, 3VIM, 3VIN, 3VIO, 3VIP
OsaBgl	β-glucosidase	*Oryza sativa Japonica* Group	Q8L7J2	3GNO, 3GNP, 3GNR, 3WBA, 3WBE
OsaBgl2	β-glucosidase	*Oryza sativa Japonica* Group	B8AVF0	3PTK, 3PTM,3PTQ
OsaBgl3	monolignol β-glucosidase	*Oryza sativa Japonica* Group	Q7XSK0	7D6A, 7D6B

OsaBgl4	β-glucosidase	*Oryza sativa Japonica* Group	Q75I93	2RGL, 2RGM, 3AHT, 3AHV, 3F4V, 3F5J, 3F5K, 3F5L, 3SCN, 3SCO, 3SCP, 3SCQ, 3SCR, 3SCS, 3SCT, 3SCU, 3SCV, 3SCW, 4QLJ, 4QLK, 4QLL, 7BZM
PchBgl	β-glucosidase	*Phanerochaete chrysosporium* K-3	Q25BW5	2E3Z, 2E40
SfrBgl	β-glycosidase	*Spodoptera frugiperda*	O61594	5CG0
ThaBgl	β-glucosidase ThBgl2	*Trichoderma harzianum*	A0A0F9XM91	5JBO
ThaBgl2	β-1,4-glucosidase	*Trichoderma harzianum* T7	A0A0F9ZQA8	5JBK
ThaBgl3	Beta-1,4-glucosidase	*Trichoderma harzianum*	A3FPG4	5BWF
ThaBgl4	Glycoside hydrolase family 1 protein	*Trichoderma harzianum* CBS 226.95	A0A2T4AR08	6EFU
HjeBgl	β-glucosidase	*Hypocrea jecorina*	G0RIF5	6KHT
HjeBgl2	β-glucosidase 2	*Hypocrea jecorina*	O93785	3AHY, 4GXP
TreBgl	β-glucosidase 2	*Trifolium repens*	P26205	1CBG
**Unclassified**				
	**Protein Name**	**Organism**	**UniProt**	**PDB**
unmicBgl	β-glucosidase	uncultured microorganism	A0A1E1FFN6	5XGZ, 7WDN, 7WDO, 7WDP, 7WDR, 7WDS, 7WDV
unidBgl	β-glucosidase	unidentified	A0A2I2LGB3	5WKA

Table S3. Table S3. Crystal structures of ligand-bound Bgl.

Name (organism)	PDB	Complex	Ligand Name	Ligand ID	Resolution (Å)
AtuBgl (*Agrobacterium tumefaciens*)	6RJK	Native			
	6RJM	Product glucose	alpha-D-glucopyranose	GLC	2.11
	6RJO	Substrate analog salicin	2-(hydroxymethyl)phenyl beta-D-glucopyranoside	SA0	1.80
	6RK2	Substrate SAG	2-(alpha-L-altropyranosyloxy)benzoic acid	6GR	2.09
PpoBgl2 (*Bacillus polymyxa*)	2O9P	Native			2.10
	2JIE	2-F-glucose	2-deoxy-2-fluoro-alpha-D-glucopyranose	G2F	2.30
	2O9R	Thiocellobiose	thio-beta-cellobiose		2.30
	2O9T	Glucose	beta-D-glucopyranose	BGC	2.15
TmaBgl (*Thermotoga maritima*)	1OD0	Native			1.9
	1OIF	5-hydroxylmethyl-3,4-dihydroxypiperidin	5-hydroxylmethyl-3,4-dihydroxypiperidin	IFM	2.12
	1OIM	1-deoxynojirimycin	1-deoxynojirimycin	NOJ	2.15
	1OIN	2-deoxy-2-fluoro-alpha-D-glucopyranose	2-deoxy-2-fluoro-alpha-D-glucopyranose	G2F	2.15
OsaBgl (*Oryza sativa* Japonica Group)	3GNO	Native			1.83
	3GNP	Octyl-beta-D-tihio-glucoside	octyl 1-thio-beta-D-glucopyranoside	SOG	1.80
	3GNR	2-deoxy-2-fluoroglucoside	2-deoxy-2-fluoro-alpha-D-glucopyranose	G2F	1.81
OsaBgl2 (*Oryza sativa*)	3PTK	Native			2.49
	3PTM	2-fluoroglucopyranoside	2-deoxy-2-fluoro-alpha-D-glucopyranose	G2F	2.40
	3PTQ	Dinitrophenyl 2-deoxy-2-fluoro-beta-D-glucopyranoside	2,4-dinitrophenyl 2-deoxy-2-fluoro-beta-D-glucopyranoside	NFG	2.45
OsaBgl3 (*Oryza sativa* Japonica Group)	7D6A	Native			1.70
	7D6B	Delta-gluconolactone	D-glucono-1,5-lactone	LGC	2.10
OsaBgl4 (*Oryza sativa* Japonica Group)	2RGL	Native			2.20
	2RGM	2-deoxy-2-fluoro-alpha-D-glucopyranose	2-deoxy-2-fluoro-alpha-D-glucopyranose	G2F	1.55
PchBgl (*Phanerodontia chrysosporium*)	2E3Z	Native			1.50
	2E40	Gluconolactone	D-glucono-1,5-lactone	LGC	1.90

References

1. Sievers, F.; Wilm, A.; Dineen, D.; Gibson, T.J.; Karplus, K.; Li, W.; Lopez, R.; McWilliam, H.; Remmert, M.; Soding, J.; et al. Fast, scalable generation of high-quality protein multiple sequence alignments using Clustal Omega. *Mol. Syst. Biol.* **2011**, *7*, 539, doi:10.1038/msb.2011.75.
2. Letunic, I.; Bork, P. Interactive Tree Of Life (iTOL) v5: an online tool for phylogenetic tree display and annotation. *Nucleic Acids Res.* **2021**, *49*, W293-W296, doi:10.1093/nar/gkab301.
